# Domain-level Identification of Single Prokaryotic Cells by Optical Photothermal Infrared Spectroscopy

**DOI:** 10.1264/jsme2.ME23052

**Published:** 2023-10-18

**Authors:** Motoko Igisu, Masayuki Miyazaki, Sanae Sakai, Satoshi Nakagawa, Hiroyuki D. Sakai, Ken Takai

**Affiliations:** 1 Super-cutting-edge Grand and Advanced Research (Sugar) Program, Institute for Extra-cutting-edge Science and Technology Avant-garde Research (X-star), Japan Agency for Marine-Earth Science and Technology (JAMSTEC), 2–15 Natsushima, Yokosuka, Kanagawa 237–0061, Japan; 2 Laboratory of Marine Environmental Microbiology, Division of Applied Biosciences, Graduate School of Agriculture, Kyoto University, Oiwake-cho, Kitashirakawa, Sakyo-ku, Kyoto 606–8502, Japan; 3 Exploratory Research Center on Life and Living Systems (ExCELLS), National Institute of Natural Sciences, 5–1 Higashiyama, Myodaiji, Okazaki, Aichi 444–8787, Japan; 4 Department of Science and Engineering for Sustainable Innovation, Faculty of Science and Engineering, Soka University, Hachioji, Tokyo, Japan; 5 Present address: BioResource Research Center, Japan Collection of Microorganisms, RIKEN, Tsukuba, Ibaraki 305–0074, Japan

**Keywords:** optical photothermal infrared spectroscopy, prokaryotes, domain-level diagnosis, aliphatic C-H bonds, membrane lipids

## Abstract

Infrared spectroscopy is used for the chemical characterization of prokaryotes. However, its application has been limited to cell aggregates and lipid extracts because of the relatively low spatial resolution of diffraction. We herein report optical photothermal infrared (O-PTIR) spectroscopy of prokaryotes for a domain-level diagnosis at the single-cell level. The technique provided infrared spectra of individual bacterial as well as archaeal cells, and the resulting aliphatic CH_3_/CH_2_ intensity ratios showed domain-specific signatures, which may reflect distinctive cellular lipid compositions; however, there was interference by other cellular components. These results suggest the potential of O-PTIR for a domain-level diagnosis of single prokaryotic cells in natural environments.

The classification of bacterial and archaeal cells and the quantification of their abundance ratios (*i.e.*, domain-level identification of cells) in microbial communities are important initial steps in environmental microbiology and biogeochemistry. Recent metagenomic approaches readily provide the identification and quantification of microbial community components at different taxonomic levels using bulk DNA assemblages extracted from certain natural microbial habitats, in addition to significant insights into possible physiological and ecological functions based on reconstructed genome sequences and gene repertoires (Simon and Daniel, 2011; Grossart *et al.*, 2020; Taş *et al.*, 2021; Sato *et al.*, 2022; Zhao and Zhang, 2022). However, the metagenome-based identification and quantification of microbial community components are generally affected by methodological biases throughout DNA extraction, amplification, and sequencing procedures, and are lacking in the morphological characteristics of component cells and the cell-cell and/or cell-substrate physical relationship in microbial communities (Szóstak *et al.*, 2022; Okazaki *et al.*, 2023; Pierella Karlusich *et al.*, 2023).

Before and even during the ongoing metagenome era in environmental microbiology, microscopic techniques enable the identification of the morphological and physiological characteristics of microbial communities at the cell level. As an example, a microscopic fluorescence *in situ* hybridization (FISH) ana­lysis targeting RNA molecules in living microbial cells remains an excellent technique for the taxonomic and functional diagnosis as well as the quantification of microbial community components in natural environments and specific enrichment cultures (Amann *et al.*, 1995; Huber *et al.*, 2018; Imachi *et al.*, 2020; Liu *et al.*, 2022; Nácher-Vázquez *et al.*, 2022; Dede *et al.*, 2023). The FISH ana­lysis is based on nucleic acid probes (FISH probes) specifically binding to target sequences. Therefore, if the sequence diversity to cover is greater, the design of FISH probes technically becomes more difficult (Nácher-Vázquez *et al.*, 2022). The recent exploration of microbial dark matter (Hedlund *et al.*, 2014; Yarza *et al.*, 2014) has unveiled a diversity of previously unidentified microbial genomes (metagenome-assembled genomes and single cell-amplified genomes) in various environments (Woyke *et al.*, 2017; Jiao *et al.*, 2021; Nayfach *et al.*, 2021; Oshiki *et al.*, 2022). It may become difficult to design universal FISH probes that target these microbial dark matter cells or domain-specific probes that deal with microbial communities including diverse archaeal and bacterial microbial dark matter (Nácher-Vázquez *et al.*, 2022). Furthermore, there are functionally inert cells and a number of inorganic and organic substances in natural samples that may prevent detection and quantitative estimations by FISH probe-specific signatures (Huber *et al.*, 2018). Therefore, a domain-level diagnosis by a microscopic FISH ana­lysis is not always applicable to the natural microbial community.

Alternatively, microscopic Raman and Fourier transform infrared (FTIR) spectroscopies are potential domain-level diagnostic techniques for prokaryotic cells (Hedrick *et al.*, 1991; Kanno *et al.*, 2021). A recent study indicated the potential of Raman microspectroscopy for the domain- to species-level classification of prokaryotes at the single-cell level when combined with a statistical ana­lysis (Kanno *et al.*, 2021). Previous studies demonstrated the applicability of FTIR spectroscopy to the domain-level classification of prokaryotic cell aggregates and cellular lipid extracts (Hedrick *et al.*, 1991; Igisu *et al.*, 2012). Hedrick *et al.* (1991) showed that specific infrared (IR) absorption bands of ester carbonyl (C=O) and aliphatic methyl (CH_3_) groups in cellular lipid extracts were useful for distinguishing between archaeal and bacterial cells. Igisu *et al.* (2012) focused on known domain-specific lipid components (*e.g.* main hydrocarbon chain species) and demonstrated that the absorbance ratio of specific IR absorption bands for aliphatic methylene (CH_2_) and aliphatic methyl (CH_3_) groups (aliphatic CH_3_/CH_2_: R_3/2_ values) may be a chemical indicator for the domain-level identification of prokaryotic cell aggregates even after chemical cell fixation (formaldehyde) and nucleic acid staining (4′,6-diamidino-2-phenylindole: DAPI) processes. However, traditional FTIR microspectroscopy cannot detect IR signals from a single prokaryote cell due to the relatively low spatial resolution of diffraction. Optical photothermal infrared (O-PTIR) spectroscopy, which is an innovative new system for an IR ana­lysis with a sub-micron spatial resolution, has recently enabled us to obtain the IR spectra of bacteria at the single-cell level in the fingerprint region (1,800–800‍ ‍cm^–1^), and successfully differentiated isotopically labeled and unlabeled bacterial cells at the single-cell level when used in combination with a statistical ana­lysis (Lima *et al.*, 2021, 2022). This new system is very promising for single-cell IR spectroscopy of archaea as well as bacteria in a wider wavenumber range; however, O-PTIR spectroscopy has not yet been applied to the domain-level diagnosis of prokaryotic cells.

We herein report O-PTIR spectroscopy of cultivated bacterial and archaeal cells for its future application to the domain-level diagnosis of individual prokaryotic cells in natural microbial communities. Ten different prokaryotic cultures (3 and 6 species of bacteria and archaea, respectively, and an archaeon with its symbiotic archaeon) were subjected to an O-PTIR ana­lysis (Table S1).

Bacterial and archaeal cells grown to the late exponential phase with standard media under optimal conditions were chemically fixed by paraformaldehyde at a final concentration of 5% (w/v). Fixed cells were washed several times with deionized distilled water (DDW) (see Supplemental material for details). Two species (*Escherichia coli* and *Archaeoglobus* sp. strain MCR1) were stained with DDW containing DAPI. Prior to the O-PTIR ana­lysis, we performed a traditional FTIR microspectroscopic ana­lysis on the fixed and stained cell assemblages to directly compare the results of O-PTIR and FTIR (see Supplemental material for details).

Representative O-PTIR spectra of individual bacterial and archaeal cells are shown in Fig. 1. The O-PTIR spectra of both bacterial and archaeal cells had specific bands at approximately 3,300‍ ‍cm^–1^ (O-H and N-H), 2,960‍ ‍cm^–1^ (aliphatic CH_3_: end-methyl), and 2,925 and 2,850‍ ‍cm^–1^ (aliphatic CH_2_: chain-methylene) (assignments are based on Naumann, 2001). The combination of single wavenumber images at 2,925‍ ‍cm^–1^ with optical images obtained under the O-PTIR microscope revealed that O-PTIR spectra were collected from individual bacterial and archaeal cells (Fig. 2). This result demonstrated that O-PTIR spectroscopy provided the IR spectra of not only bacterial cells, but also archaeal cells in the 3,600–2,700‍ ‍cm^–1^ region at the single-cell level. The R_3/2_ values of individual bacterial and archaeal cells on O-PTIR spectra were calculated and compared with those of bacterial and archaeal cell assemblages obtained by traditional FTIR microspectroscopy (micro-FTIR) (Fig. 3 and Table S1). Except for the fixed and stained cells of *Archaeoglobus* sp. strain MCR1, all R_3/2_ values were similar within the same domain, but distinct between the different domains (*P*<0.05) (Fig. 3 and Table S1). These results were consistent with those obtained by traditional FTIR microspectroscopy (Fig. 3). The R_3/2_ values of *Archaeoglobus* sp. strain MCR1 markedly different between O-PTIR and micro-FTIR ana­lyses (Table S1). Since *Archaeoglobus* sp. strain MCR1 is a sulfate-reducing archaeon, cultures contained many metal sulfide minerals in addition to cells. It was not possible to remove sulfide minerals by washing with DDW and they may have adsorbed certain types and amounts of organic matter. These organic compounds adsorbed with sulfide minerals may have affected the R_3/2_ value estimation of *Archaeoglobus* sp. strain MCR1 cell assemblages by the micro-FTIR ana­lysis (Table S1). Although there was only one example tested in the present study, the R_3/2_ value obtained by O-PTIR spectra was not affected by the DAPI staining of cells; mean values for fixed and fixed-and-stained *E. coli* cells were 0.68±0.03 (*n*=10) and 0.66±0.04 (*n*=10), respectively (Table S1). These results suggest that the R_3/2_ values measured using O-PTIR spectra were effective for a domain-level diagnosis of individual microbial cells, as previously reported by traditional FTIR microspectroscopy, even after formaldehyde fixation and DAPI staining.

To test the spatial resolution of O-PTIR, we analyzed an archaeon species (*Metallosphaera* sp. strain AS-7; 0.7–1.4‍ ‍μm in diameter) co-existing with small cells of its symbiotic archaeon (*Microcaldus variisymbioticus* strain ARM-1; 240–440‍ ‍nm in diameter) belonging to the DPANN superphylum as an example of recently identified microbial dark matter (Sakai *et al.*, 2022) (Table S1 and Fig. S1). We identified possible symbiont and host cells using optical microphotographs and then conducted O-PTIR spectroscopy on the specific locations of possible symbiont and host cells. The R_3/2_ values of *Metallosphaera* sp. AS-7 (host) with *M. variisymbioticus* strain ARM-1 (symbiont) were 0.92±0.07 (*n*=3) and 0.88±0.06 (*n*=4), respectively. However, O-PTIR imaging at 2,925‍ ‍cm^–1^ and a single-point O-PTIR ana­lysis at a location without apparent cells on the CaF_2_ surface revealed similar spectroscopic signatures to those of cells, and the R_3/2_ value of the CaF_2_ surface without cells was 0.70 (*n*=1) (Fig. S1). This result appeared to be due to organic matter derived from cells that burst during sample preparation. Since the measurement number was limited, a statistical ana­lysis was not conducted in the present study. Nevertheless, the results obtained are consistent with the mean R_3/2_ values (bulk ana­lysis) obtained by traditional FTIR microspectroscopy (0.90±0.01 for *Metallosphaera* sp. AS-7 with *M. variisymbioticus* strain ARM-1) (Table S1), and the R_3/2_ values of the *Metallosphaera* or *Microcaldus* strain were consistent with those of cells from a representative archaeal species (0.95±0.07) (Igisu *et al.*, 2012).

It is important to note some exceptional results and issues to consider for a domain-level diagnosis based on the R_3/2_ values of FTIR and O-PTIR microspectroscopy. The R_3/2_ values of some bacterial cells were higher than the reported mean values for most of the bacterial cells (0.65±0.07) (Igisu *et al.*, 2012). In our experiments, exceptions were observed in two cases. The first case was the cells of *Bacillus subtilis*, which showed IR spectra with/without a strong ~1,740‍ ‍cm^–1^ band (Fig. S2A). The R_3/2_ value for *B. subtilis* with the ~1,740‍ ‍cm^–1^ band in its IR spectra was 0.83±0.01 (*n*=5), while that for *B. subtilis* without the ~1,740‍ ‍cm^–1^ band was 0.68±0.05 (*n*=5). The strong ~1,740‍ ‍cm^–1^ band is derived from C=O bonds and is considered to represent high concentrations of poly-β-hydroxybutyrate (PHB) in cells as the storage material (El-Kadi *et al.*, 2021). Therefore, PHB-enriched bacterial cells revealed higher R_3/2_ values due to the relative abundance of end-methyl to chain-methylene in PHB. However, this case may be recognized by examining spectra in the 1,800–1,700‍ ‍cm^–1^ region, and observations in a wide range of wavenumbers are helpful for the precise domain-level diagnosis of prokaryotic cells by O-PTIR and FTIR. The second case is *Clostridium* sp. or *Ilyobacter* sp. cells, which showed R_3/2_ values of 0.91±0.02 (*n*=10) and 0.89±0.10 (*n*=10), respectively, with no marked differences being observed in their IR spectral patterns from those of other bacterial species (Fig. S2B). Bacterial cells at different stages of the life cycle show distinct IR spectra (Johnson *et al.*, 2009). The exceptional R_3/2_ values of some bacterial species may be associated with the enrichment and depletion of specific cellular materials at different stages of the cell and life cycles and, thus, this needs to be considered when a domain-level diagnosis by FTIR or O-PTIR microspectroscopy is applied to microbial cells in natural communities. Another limitation of the present study is that highly sensitive O-PTIR imaging and a single-point O-PTIR ana­lysis of prokaryotic cells detected signatures not only from cells, but also from extracellular organic matter (including the potential leakage of intracellular organic matter) on the CaF_2_ substrate. As an example, the O-PTIR results of *Archaeoglobus* sp. strain MCR1 cells are shown in Fig. S3. Although cell assemblages were fixed and thoroughly washed with DDW during sample preparation (see Supplemental material), it was difficult to completely remove extracellular organic matter (*e.g.*, water-insoluble cell debris and polysaccharides) from cell assemblage samples in some cases (*e.g.*, Fig. S1 and S3). Therefore, it is important to collect the single-point O-PTIR spectra of target cells by checking both optical and O-PTIR images in order to avoid interference from extracellular organic matter on the CaF_2_ substrate.

In summary, we confirmed the resolution and sensitivity of O-PTIR spectroscopy to obtain the IR spectra of individual bacterial and archaeal cells, and demonstrated that the resulting R_3/2_ values may be used for a domain-level diagnosis of prokaryotes at the single-cell level, even though the new technique will be applied with some caution. O-PTIR spectroscopy provides IR spectra from isotopically-labeled enrichment bacterial cells in the fingerprint wavenumber region (1,800–800‍ ‍cm^–1^) (Lima *et al.*, 2021, 2022). In consideration of spectroscopic characteristics covering a wider range of wavenumbers, microscopic O-PTIR spectroscopy has potential in future environmental microbiology investigations, such as not only the domain-level diagnosis and quantification of microbial communities, but also the characterization of physiological and metabolic functions at the single-cell level when used in combination with isotope-tracer cultivations.

## Citation

Igisu, M., Miyazaki, M., Sakai, S., Nakagawa, S., Sakai, H. D.., and Takai, K. (2023) Domain-level Identification of Single Prokaryotic Cells by Optical Photothermal Infrared Spectroscopy. *Microbes Environ ***38**: ME23052.

https://doi.org/10.1264/jsme2.ME23052

## Supplementary Material

Supplementary Material

## Figures and Tables

**Fig. 1.**
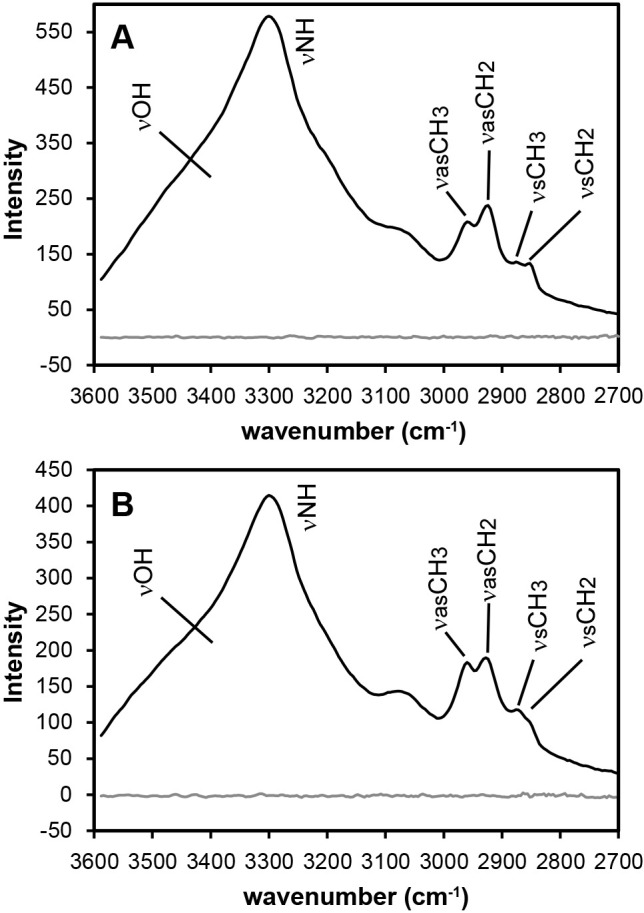
O-PTIR spectra for prokaryotic cells and a CaF_2_ substrate. (A) indicates the results of *Escherichia coli* cells, and (B) indicates the results of *Archaeoglobus fulgidus* cells. Bands were observed around 3,300‍ ‍cm^–1^ (NH and OH bonds), 2,960‍ ‍cm^–1^ (asymmetric aliphatic CH_3_: end-methyl), and 2,925‍ ‍cm^–1^ and 2,850‍ ‍cm^–1^ (asymmetric and symmetric aliphatic CH_2_, respectively: chain-methylene).

**Fig. 2.**
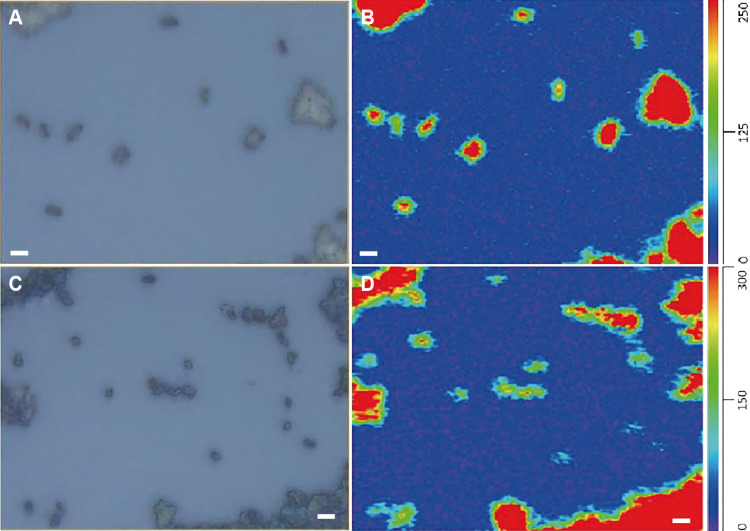
O-PTIR imaging of prokaryotic cells. Optical images of *Escherichia coli* (A) and *Archaeoglobus fulgidus* (C) cells obtained using the O-PTIR microscope. Spatial distribution of intensity at 2,925‍ ‍cm^–1^ collected using O-PTIR imaging of cells of *E. coli* (B) and *A. fulgidus* (D). Scale bars indicate 2‍ ‍μm. Color scales: red indicates higher intensity, blue lower intensity.

**Fig. 3.**
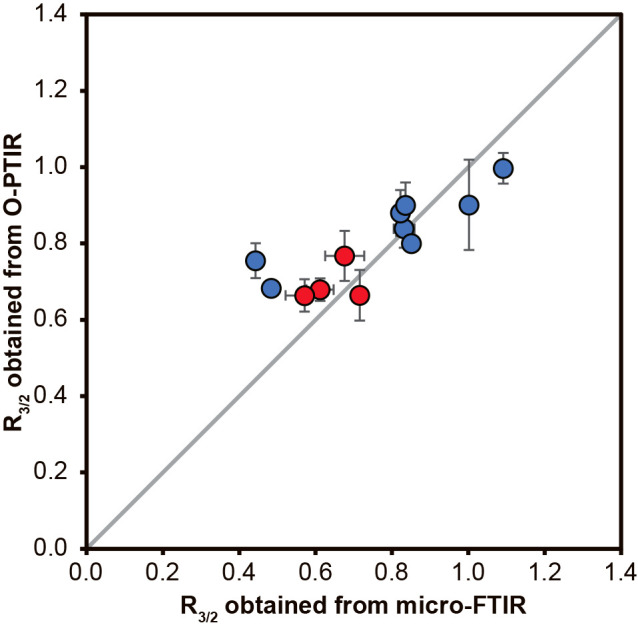
Relationship between R_3/2_ values for prokaryotic cells obtained from traditional micro-FTIR ana­lyses (X-axis) and O-PTIR ana­lyses (Y-axis). Data points for Archaea are represented in blue, while those for Bacteria are shown in red.
