# Supplementary material for: Domain-level Identification of Single Prokaryotic Cells by Optical Photothermal Infrared Spectroscopy
Source: Microbes Environ. 2023 Oct 18;38(4):ME23052. doi: 10.1264/jsme2.ME23052 (PMC10728636; doi:10.1264/jsme2.ME23052)
Supplement: Supplementary file 1 — Supplementary Material [file 38_23052_s1.pdf]

Supplemental material

Materials and methods

Bacterial and archaeal cultures and preparation for O-PTIR spectroscopy and FTIR microspectroscopy

Bacterial and archaeal strains used in this study are indicated in Table S1. *Escherichia coli* K-12, *Deinococcus radiodurans* JCM16871^{T}, *Methanoculleus marisnigri* JR1^{T} and *Archaeoglobus fulgidus* JCM9628^{T} were purchased from culture collections. Some of the bacterial and archaeal strains were originally isolated by coauthors from various extreme environments. *Bacillus subtilis* YTA-0001, *Streptomyces* sp. strain No.17, *Methanosarcina mazei* NT-MS1, *Ilyobacter* sp. strain KT, and *Clostridium* sp. strain KT were isolated from the deep sea sediments of Mariana Trench, Suruga Bay, Japan Trench, forearc basin of Japan Trench, respectively. *Archaeoglobus* sp. strain MCR1 was isolated from a deep-sea hydrothermal environment. The information of *Metallosphaera* sp. strain AS-7, and *Microcaldus variisymbioticus* ARM-1^{T} was described in Sakai *et al*. (2020).

All the bacterial and archaeal species and strains were cultivated with the optimal media under optimal conditions. The late exponential growth phase of culture was chemically fixed by a media containing 4% (w/v) paraformaldehyde for 3 hours to overnight. The fixed cells were harvested by centrifugation at 4˚C under an air atmosphere. The harvested cells were washed with PBS (phosphate-buffered saline) for 3 times, then washed three times with deionized distilled water (DDW). Finally, cell pellets were placed on the CaF_2 disks and then were completely dried up (fixed cells). In addition, before mounting on the CaF_2 disks, some of the pellets were stained with DDW containing 4', 6-diamidino-2-phenylindole (DAPI) (10 µg/ml). After staining with DAPI, the cells were washed with DDW three times, placed on the CaF_2 disks and dried (stained cell).

Micro-FTIR measurement

FTIR measurements of bacterial and archaeal cell assemblages were conducted using an FTIR micro-spectrometer equipped with narrow band mercury cadmium telluride detector (JASCO, FTIR6200+IRT7000). A reference background spectrum was first measured at a place away from the mounted sample (CaF_2 alone), and then transmission IR spectrum of sample was measured. Five points were measured within the same sample, respectively (one exception is stained *A. lithotrophicus*: n=1) (Table S1). Rectangular aperture size of the analysis was 50 µm × 50 µm.

Sixty four scans were accumulated at 4 cm^{-1} spectral resolution in a range from 4000 to 1000 cm^{-1}. The IR spectral data were analyzed with a software program (JASCO, Spectra Manager).

O-PTIR measurement

O-PTIR measurement of individual bacterial and archaeal cells were conducted by using a mIRage microscope (Photothermal Spectroscopy Corp., Santa Barbara, USA) at Nihon Thermal Consulting Co., Ltd. (Japan). O-PTIR imaging analysis (step size: 200 nm) at 2925 cm^{-1} corresponding to aliphatic CH_2 group were first performed in reflection mode to examine spatial distributions of organic matter. A tunable optical parametric oscillator (OPO) laser was used as the IR pump beam, whereas the probe beam was a continuous wave visible (532 nm) laser. Then, single-point O-PTIR spectra were collected over the spectral region of ~3600-2700 cm^{-1} with an avalanche photodiode detector. Spectra were recorded with a spectral resolution of 4 cm^{-1}, and averages of 2 to 10 to obtain enough signal-to-noise ratio. The IR and visible laser powers were optimized using neutral density (ND) filters to avoid any laser damages and signal saturation. The probe laser power was set to 0.2 %, or 0.4 % of its maximum power of 200 mW. The IR pump laser power was adjusted using single ND filter of 24 % or double ND filters (79 % ND filter and an additional germanium ND filter with optical density 0.3) of its maximum power up to 200 mW depending on the wavenumber. Although the IR laser power was set slightly higher, there was no change in the optical image before and after the measurement and no significant change in signal intensity during spectral integration, suggesting that there was no sample damage caused by the laser. Since the sample drifted several hundred nm in about one hour of measurement time, we did not perform hyperspectral analysis, but instead checked for the presence of organic material at an intensity of 2925 cm^{-1} and then performed a spot analysis. Two to seventeen cells were measured within the same sample, respectively (Table S1).

$R_{3/2}$ values

In order to evaluate the spectral characteristics, we introduced the aliphatic CH_3/CH_2 absorbance ratio ($R_{3/2}$):

$$R_{3/2} = [\nu_{as}CH_3]/ [\nu_{as}CH_2] \qquad (1)$$

where $[\nu_{as}CH_3]$ and $[\nu_{as}CH_2]$ represented peak heights of asymmetric stretching bands for aliphatic CH_3 (end-methyl; ~2960 cm^{-1}) and CH_2 (chain-methylene; ~2925 cm^{-1}) after linear baseline correction, respectively (Igisu *et al.*, 2012).

Statistical analysis

The $R_{3/2}$ values in the text are shown as mean ± SD. Two-group comparisons (domain bacteria and archaea) of the mean $R_{3/2}$ values were performed using the Mann-Whitney test because of small sample sizes. A *P* value below 0.05 was considered statistically significant.

References

Igisu, M., Takai, K., Ueno, Y., Nishizawa, M., Nunoura, T., Hirai, M., *et al.*. (2012) Domain-level identification and quantification of relative prokaryotic cell abundance in microbial communities by micro-FTIR spectroscopy. *Environ. Microbiol Rep* **4**: 42-49.

Sakai, H. D., Nur, N., Kato, S., Yuki, M., Shimizu, M., Itoh, T., *et al.* (2022) Insight into the symbiotic lifestyle of DPANN archaea revealed by cultivation and genome analyses. *Proc Natl Acad Sci USA* **119**: e2115449119.

Table S1. $R_{3/2}$ values and numbers of analyses of different prokaryotic cells.

Sample	Micro-FTIR		O-PTIR (mIRage)	
	$R_{3/2}$	*number*	*$R_{3/2}$*	*number*
Bacteria				
Escherichia coli K-12	0.61±0.04	5	0.68±0.03	10
Escherichia coli K-12 (DAPI stained)	0.57±0.05	5	0.66±0.04	10
Bacillus subtilis YTA-0001	0.68±0.05	5	0.77±0.07	8
Streptomyces sp. strain No.17	0.72±0.01	5	0.66±0.07	4
Deinococcus radiodurans JCM16871^T	0.77±0.04	5	–[a]	–
mean ± SD (except for DAPI stained *E. coli*)	0.69±0.07		0.70±0.06	
Archaea				
Aeropyrum camini	0.82±0.02	5	0.88±0.06	11
Methanoculleus marisnigri JR1^T	1.00±0.01	5	0.90±0.12	6
Methanosarcina mazei NT-MS1	1.09±0.01	5	1.00±0.04	11
Archaeoglobus sp. strain MCR1	0.48±0.00	5	0.68±0.01	2
Archaeoglobus sp. strain MCR1 (DAPI stained)	0.44	1	0.75±0.05	17
Archaeoglobus fulgidus JCM9628^T	0.83±0.03	5	0.84±0.05	9
Metallosphaera sp. strain AS-7	0.85±0.04	5	0.80±0.02	4
Microcaldus variisymbioticus ARM-1^T+ *Metallosphaera* sp. strain AS-7	0.84±0.02	5	0.90±0.06	9
mean ± SD (except for DAPI stained *Archaeoglobus* sp. strain MCR1)	0.85±0.19		0.86±0.10	

Errors show standard deviation (SD).

T; type strain.

[a]Not analyzed.

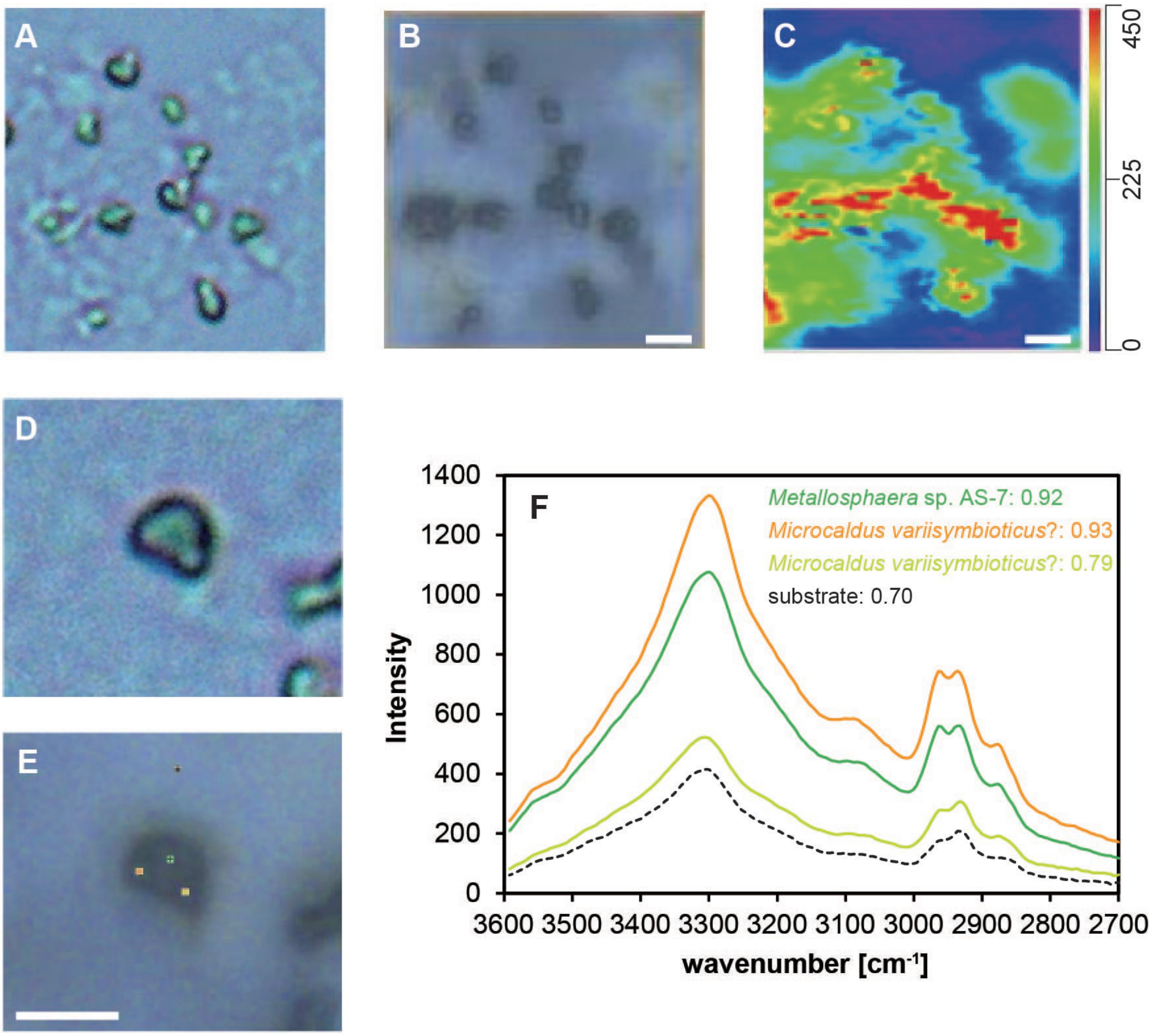

Figure S1. O-PTIR images and spectra of *Metallosphaera* sp. strain AS-7 with *Microcaldus variisymbioticus* strain ARM-1. (A) shows the optical microphotograph of *Metallosphaera* sp. strain AS-7 with *M. variisymbioticus* strain ARM-1 cells. (B) shows the optical image of *Metallosphaera* sp. strain AS-7 with *M. variisymbioticus* strain ARM-1 taken by O-PTIR microscope. (C) shows the spatial distribution of the intensity at 2925 cm^{-1} collected using O-PTIR imaging of *Metallosphaera* sp. strain AS-7 with *M. variisymbioticus* strain ARM-1. The color scales represent the higher peak height (red) and the lower one (blue) in intensity. (D) shows the magnified optical microphotograph in which *M. variisymbioticus* cells attach the host cells and (E) shows the optical image obtained by using O-PTIR microscope at the same areas. Scale bars indicate 2 µm. (F) shows the O-PTIR spectra with $R_{3/2}$ values of cells (green from a cell of *Metallosphaera* sp. strain AS-7, orange, and yellow from cells of possibly *M. variisymbioticus*, and dotted black from CaF_2 surface without cell). Analytical spots are shown in (E).

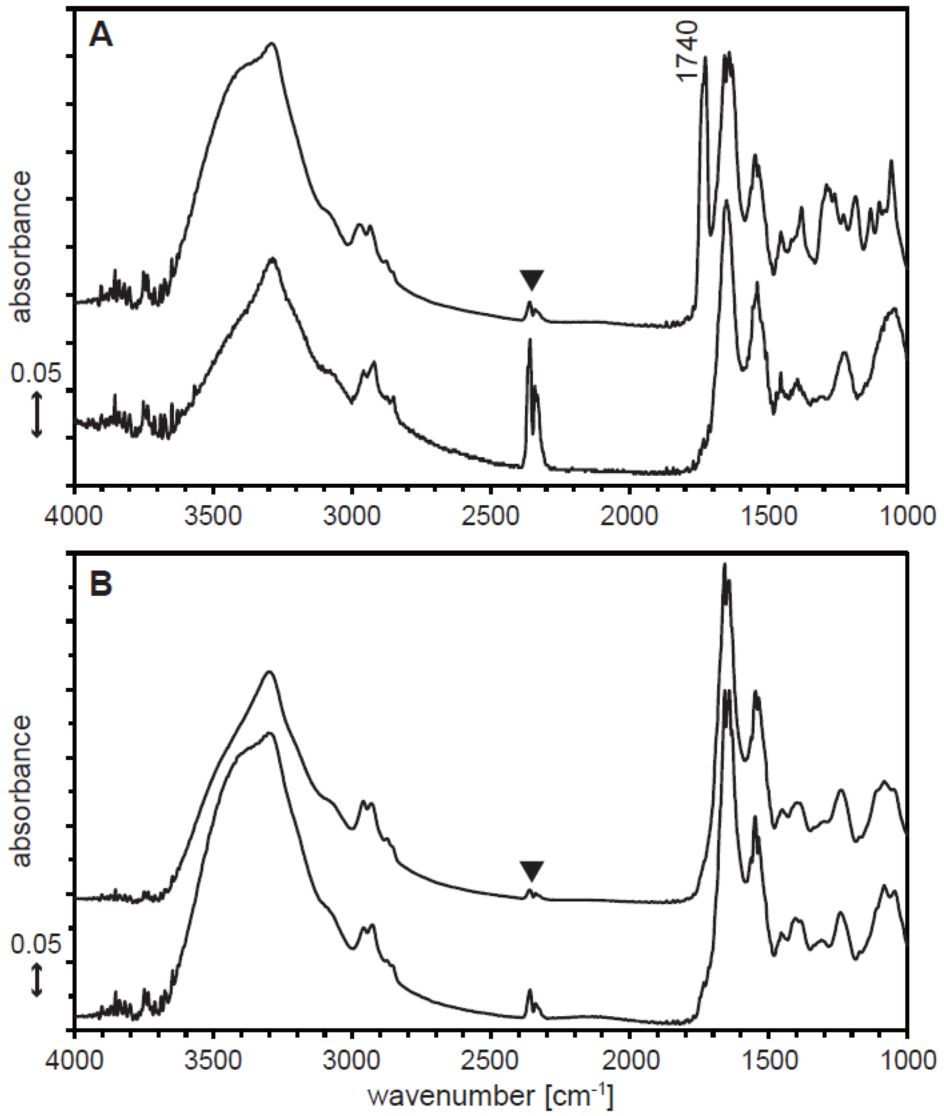

Figure S2. IR spectra with higher $R_{3/2}$ values (top) and with mean $R_{3/2}$ values (bottom) obtained by traditional FTIR microspectroscopy. (A) shows *Bacillus subtilis* cells, and (B) shows *Clostridium* sp. cells. The bands around 3300 cm^{-1} (NH and OH bonds), 2960, 2925 and 2850 cm^{-1} (aliphatic CH), 1650 and 1540 cm^{-1} (C=O: amide I, and CNH: amide II, respectively) are observed. The ~1740 cm^{-1} band is derived from C=O bonds in poly-β-hydroxybutyrate. The IR spectrum around 2300 cm^{-1} (filled triangles) represents the absorption by CO_2 in the air.

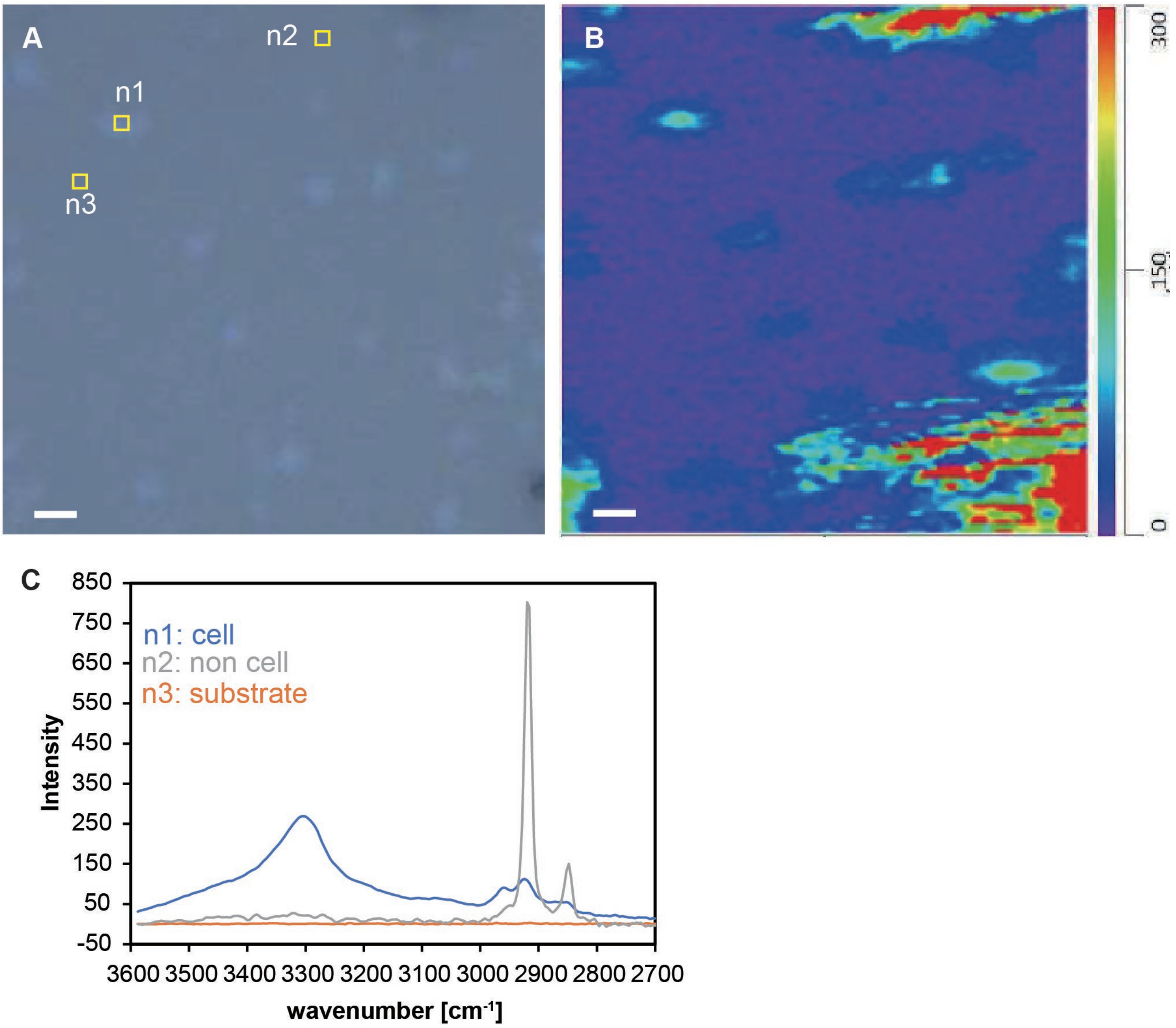

Figure S3. O-PTIR image and spectra of cells of *Archaeoglobus* sp. strain MCR1. (A) shows the optical image obtained by using O-PTIR microscope. Squares are analyzed area shown in (C). (B) shows the spatial distribution of the intensity at 2925 cm^{-1} collected using O-PTIR imaging. The color scales represent the higher peak height (red) and the lower one (blue) in intensity. (C) shows the O-PTIR spectra of cell, non-cell, and untreated CaF_2 surface. Scale bars indicate 2 μm.
